# Clinicopathological Significance of Minimal Extrathyroid Extension in Solitary Papillary Thyroid Carcinomas

**DOI:** 10.1245/s10434-015-4659-0

**Published:** 2015-06-16

**Authors:** Chang Gok Woo, Chang Ohk Sung, Yun Mi Choi, Won Gu Kim, Tae Yong Kim, Young Kee Shong, Won Bae Kim, Suck Joon Hong, Dong Eun Song

**Affiliations:** Department of Pathology, Asan Medical Center, University of Ulsan College of Medicine, Seoul, Korea; Department of Internal Medicine, Asan Medical Center, University of Ulsan College of Medicine, Seoul, Korea; Department of Surgery, Asan Medical Center, University of Ulsan College of Medicine, Seoul, Korea

## Abstract

**Background:**

The definitive diagnosis of minimal extrathyroid extension (ETE) is subjective because a well-defined true capsule is absent in the thyroid gland. We subclassified the extent of minimal ETE and investigated the clinicopathological significance of the presence of minimal ETE in patients with solitary papillary thyroid carcinomas (PTCs) and solitary papillary thyroid microcarcinomas (PTMCs).

**Methods:**

A series of 546 patients with solitary PTCs, including 144 patients with solitary PTMCs, were retrospectively analyzed. Whether the presence of minimal ETE had an effect on recurrence-free survival (RFS) along with other clinicopathological parameters was investigated.

**Results:**

The only independent prognostic factor found to be associated with recurrence was the presence of LN metastasis in solitary PTC (*p* = 0.002) but not in solitary PTMC groups (*p* = 0.073). The presence of minimal ETE had no effect on RFS in both solitary PTC (*p* = 0.053) and solitary PTMC (*p* = 0.816).

**Conclusions:**

The presence of minimal ETE has no significant influence on RFS in solitary PTC and PTMC. There is a risk of overrepresenting the T3 category in solitary PTC and PTMC patients with minimal ETE.

**Electronic supplementary material:**

The online version of this article (doi:10.1245/s10434-015-4659-0) contains supplementary material, which is available to authorized users.

Papillary thyroid carcinoma (PTC) is the most common well-differentiated thyroid carcinoma and is characterized by follicular differentiation and distinct atypical nuclear features. PTC accounts for approximately 85–90 % of differentiated thyroid carcinomas.^1^ Furthermore, the incidental detection of papillary thyroid microcarcinoma (PTMC), with a maximum diameter of 10 mm or less, has recently increased as a consequence of the frequent use of ultrasonography in regular health checkup.^2^–^5^

The presence of minimal extrathyroid extension (ETE) in PTCs is defined as when tumor cells extend to the sternothyroid muscle or perithyroid soft tissue.^6^ Most cases of PTCs with minimal ETE exhibit an extension to the perithyroid soft tissue rather than to the sternothyroid muscle. The presence of a minimal ETE is classified as the T3 category in PTCs, irrespective of tumor size, by the American Joint Commission on Cancer (AJCC) cancer staging system (7th edition).^6^ Because clinical recommendations for proper treatment can vary according to the TNM stage, accurate T categorization is very important.^7^–^9^ However, histological confirmation of the presence of minimal ETE in PTCs can be both controversial and subjective among endocrine pathologists because of the absence of a well-defined true capsule in the thyroid gland.^10^–^12^ The thyroid capsule is usually composed of inconspicuous thin fibrous tissues with a variable amount of adipose tissue, blood vessels, and skeletal muscle. It is deficient in the anterior midline of the isthmus, and skeletal muscle can even be mixed with thyroid follicles within the thyroid parenchyma of this area.^10^–^12^

We classified the extent of minimal ETE and investigated the clinicopathological significance of the presence of minimal ETE to identify any effect on recurrence-free survival (RFS) in patients with solitary PTCs and PTMCs.

## Materials and Methods

### Patients

We retrospectively reviewed 546 patients (484 female, 62 male) with a solitary PTC without evidence of extensive ETE (T4 category). Distant metastases were initially detected according to the current AJCC cancer staging system in patients who underwent total thyroidectomy or hemithyroidectomy, irrespective of cervical lymph node (LN) dissection, at Asan Medical Center, Seoul, Korea, from 1998 to 2003. Patient ages ranged from 8 to 87 years (average 44 years). LN dissection was performed when a LN metastasis was clinically suspicious by ultrasonography (US) and computed tomography (CT) or was histologically confirmed by previous fine needle aspiration biopsy prior to surgery. We reviewed the patient clinical and pathological parameters, including age, gender, procedures, mean tumor diameter, extent of tumor extension, cervical LN metastasis status, and the presence of tumor recurrence (Table 1). This study was approved by the institutional review board of Asan Medical Center.

**Table 1:** Demographic and clinicopathologic characteristics of 546 patients with solitary papillary thyroid carcinoma

Characteristics	Number (%)
Age (year)	
Mean at diagnosis (range)	44 (8–87)
<45	275 (50.4)
≥45	271 (49.6)
Sex	
Female	484 (88.6)
Male	62 (11.4)
Procedures	
Lobectomy/less than total resection	95 (17.4)
Total thyroidectomy	451 (82.6)
No lymph node dissection	5 (0.9)
Central lymph node dissection	466 (85.3)
Modified radical lymph node dissection	75 (13.7)
Tumor size (mm)	
Mean (range)	20 (1–100)
≤10	144 (26.4)
>10	402 (73.6)
Extension	
Confinement to thyroid parenchyma	196 (35.9)
Minimal extrathyroid extension	350 (64.1)
Perithyroid soft tissue	259 (47.4)
Sternothyroid muscle	91 (16.7)
Cervical lymph node metastasis	
Present	334 (61.2)
Absent	212 (38.8)
Recurrence	
Present	61 (11.2)
Median duration (mo, interquartile range)	39 (16–72)
Site	
Neck level I lymph node	1 (1.6)
Neck level II, III, IV lymph nodes	50 (81.9)
Neck level V lymph node	11 (18.0)
Neck level VI lymph node	3 (4.9)
Previous resection sites	12 (19.7)
Lung (distant metastasis)	4 (6.6)
Inguinal lymph node (distant metastasis)	1 (1.9)

### Pathological Evaluation

All slides from 546 patients were reviewed by two pathologists, including one experienced endocrine pathologist (D.E.S.). We subclassified the extent of tumor extension into the following three categories: confinement to the thyroid parenchyma (E0), extension to the perithyroid soft tissue (E1), and extension to the sternothyroid muscle (E2). Among 546 patients, 196 (35.9 %) were classified as E0, 259 (47.4 %) were classified as E1, and 91 (16.7 %) were classified as E2 (Table 1). Among 546 patients, 334 patients revealed cervical lymph node metastasis at the first operation. We analyzed the extent of the lymph node metastasis at the first operation, which included maximum metastatic tumor size, N stage, and presence of extranodal extension (Supplementary Table S1).

### Follow-up

All patients with PTCs were regularly followed up every 6–12 months with physical examinations, serum thyroglobulin and anti-thyroglobulin antibody measurements, and US neck examinations. A diagnostic whole body scan was performed after total thyroidectomy and remnant RAI ablation, as previously described.^13^ Following hemithyroidectomy, a CT scan and/or 18F-FDG PET were used to detect recurrence or distant metastasis if clinically suspected. The median follow-up period for patients was 113 (range 1–168) months. Recurrence was defined as structural disease recurrence, such as the reappearance of a pathologically confirmed malignant tissue and/or the appearance of a metastatic lesion in other organs by imaging studies during follow-up.

### Statistical Analyses

The Chi squared test and Fisher’s exact test for univariate analysis were used along with binary logistic regression and Cox–Hazard regression models for multivariate analysis. To estimate survival rates, survival curves were generated using the Kaplan–Meier method and log-rank test, respectively. We used SPSS software version 18.0 and R statistical software for these analyses. Any *p* value <0.05 was considered to indicate a statistically significant difference.

## Results

### Recurrence-Free Survival Outcomes Associated with the Subclassification of an Extrathyroid Extension

Recurrence (median duration, 39 months; interquartile range, 16–72 months) occurred in 61 (11.2 %) of our patients. Recurrence was identified in diverse sites, including one or more sites and occurred in 3 central cervical LNs (level VI), 62 lateral cervical LNs (level I, II, III, IV, or V), 5 distant metastasis (inguinal LN, 1 case; lung, 4 cases), and 12 previous resection sites (Table 1). Regarding previous resection sites, there were 11 ipsilateral soft tissue (operation bed) after total thyroidectomy, which included 6 clear resection margins and 5 involved margins, and 1 contralateral remnant thyroid parenchyma after lobectomy (Supplementary Table S2).

In the primary PTCs of 61 patients with recurrence, 12 (6.1 %) were confined to the thyroid parenchyma (E0), 38 (14.7 %) extended to the perithyroid soft tissue (E1), and 11 (12.1 %) extended to the sternothyroid muscle (E2). There was a more statistically significant difference in the RFS outcomes when two groups (E0 versus E1 + E2) were analyzed than when three groups were analyzed (E0 vs. E1 vs. E2) using the log-rank test with Cox-proportional hazard analysis (Table 2; Supplementary Fig. S1). In another two-group comparison (E0 + E1 vs. E2), the presence of extension to the sternothyroid muscle had no significant effect on RFS. Therefore, we used a two-tier classification scheme of minimal ETE (E0 vs. E1 + E2) based on the AJCC cancer staging system in the subsequent evaluations of the clinicopathological significance of minimal ETE in patients with solitary PTCs.

**Table 2:** Recurrence according to subclassification of invasion in solitary papillary thyroid carcinoma

Classification	Recurrence, no. (%)	HR	95 % CI	*p* value
I				
E0	12 (6.1)	1 (ref)		0.015
E1	38 (14.7)	2.444	1.277–4.676	0.007
E2	11 (12.1)	2.076	0.916–4.786	0.080
II (current T stage)				
E0	12 (6.1)	1 (ref)		
E1 + E2	49 (14.0)	2.350	1.250–4.419	0.006
III				
E0 + E1	50 (11.0)	1 (ref)		
E2	11 (12.1)	1.144	0.595–2.198	0.686

*E0* confinment to thyroid parenchyma, *E1* extension to perithyroid soft tissue, *E2* extension to sternothyroid muscle

### Clinicopathological Parameters Associated with the Presence of a Minimal ETE

In 546 patients with solitary PTCs, there was no significant difference in the presence of minimal ETE (E1 + E2) according to gender (*p* = 0.416). Older age (odds ratio [OR] 1.688; 95 % confidence interval [CI] 1.154–2.469; *p* = 0.007), larger tumor size (OR 3.078; 95 % CI 2.040–4.645; *p* < 0.001), and the presence of a LN metastasis (OR 2.071; 95 % CI 1.407–3.048; *p* < 0.001) were significantly correlated with the presence of a minimal ETE (Supplementary Table S3).

### Clinicopathological Parameters that Affect Recurrence-Free Survival

In 546 patients with solitary PTCs, females exhibited a lower rate of recurrence (hazard ratio [HR] 0.536; 95 % CI 0.288–0.997; *p* = 0.049). A larger tumor diameter was significantly correlated with a higher recurrence rate (*p* = 0.019) in univariate analysis but not in multivariate analysis (HR 1.616; 95 % CI 0.757–3.451; *p* = 0.215). There was a significant difference between types of surgery in univariate analysis (*p* = 0.020) but not in multivariate analysis (*p* = 0.356). The presence of cervical LN metastasis was significantly associated with recurrence (HR 3.173; 95 % CI 1.536–6.553; *p* = 0.002). Among the extent of LN metastasis, N stage, presence of extranodal extension, and maximum metastatic tumor size were significantly associated with recurrence in univariate analysis, but N stage had no effect in multivariate analysis (Supplementary Table S4). Additionally, the presence of minimal ETE was significantly correlated with recurrence in univariate analysis (*p* = 0.008); however, there was no significance in multivariate analysis (HR 1.879; 95 % CI 0.992–3.560; *p* = 0.053; Table 3). Minimal ETE according to types of surgery had also no effect on recurrence (Supplementary Table S5).

**Table 3:** Clinicopathologic parameters associated with recurrence of solitary papillary thyroid carcinomas

Parameters	HR	Univariate		HR	Multivariate	
		95 % CI	*p* value		95 % CI	*p* value
Age (year)						
<45	1 (ref)			1 (ref)		
≥45	0.626	0.373–1.050	0.076	0.670	0.397–1.131	0.134
Sex						
Male	1 (ref)			1 (ref)		
Female	0.409	0.221–0.754	0.004	0.536	0.288–0.997	0.049
Procedures						
Lobectomy/less than total resection	1 (ref)			1 (ref)		
Total thyroidectomy	3.973	1.244–12.685	0.020	1.798	0.517–6.259	0.356
Size (mm)						
≤10	1 (ref)			1 (ref)		
>10	2.348	1.159–5.128	0.019	1.445	0.663–3.151	0.355
Cervical lymph node						
No metastasis	1 (ref)			1 (ref)		
Metastasis	3.932	1.938–7.978	<0.001	3.173	1.536–6.553	0.002
Invasion						
Confinement	1 (ref)			1 (ref)		
Minimal extrathyroid extension	2.350	1.250–4.419	0.008	1.879	0.992–3.560	0.053

**Fig. 1:**
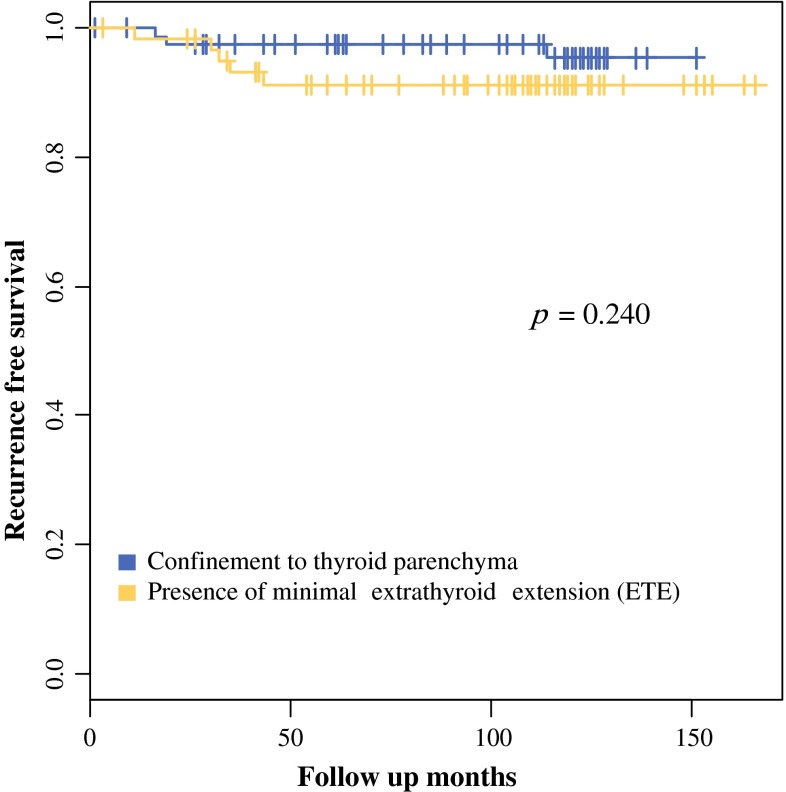
Recurrence-free survival (RFS) according to presence of minimal ETE in solitary PTMCs. There was no significant difference in the RFS between the absence and presence of minimal ETE in solitary PTMCs (*p* = 0.240)

In 144 patients with solitary PTMCs, the presence of minimal ETE was not associated with tumor recurrence in univariate analysis (*p* = 0.254) and multivariate analysis (HR 1.215; 95 % CI 0.235–6.278; *p* = 0.816; Table 4). Additionally, there was no significant difference in RFS outcomes between the absence and presence of minimal ETE (*p* = 0.240; Fig. 1). The presence of cervical LN metastasis was significantly associated with recurrence in univariate analysis (*p* = 0.034), but there was no significant difference in multivariate analysis (HR 7.353; 95 % CI 0.831–65.051; *p* = 0.073; Table 4).

**Table 4:** Clinicopathologic parameters associated with recurrence of solitary papillary thyroid microcarcinomas

Parameters	HR	Univariate		HR	Multivariate	
		95 % CI	*p* value		95 % CI	*p* value
Age (year)						
<45	1 (ref)			1 (ref)		
≥45	0.250	0.051–1.241	0.090	0.324	0.062–1.702	0.183
Sex						
Male	1 (ref)			1 (ref)		
Female	0.417	0.051–3.404	0.414	0.905	0.108–7.594	0.927
Procedures						
Lobectomy/less than total resection	1 (ref)			1 (ref)		
Total thyroidectomy	2.410	0.486–11.947	0.281	1.277	0.213–7.673	0.789
Cervical lymph node						
No metastasis	1 (ref)			1 (ref)		
Metastasis	9.599	1.181–78.042	0.034	7.353	0.831–65.051	0.073
Invasion						
Confinement	1 (ref)			1 (ref)		
Minimal extrathyroid extension	2.303	0.550–9.643	0.254	1.215	0.235–6.278	0.816

## Discussion

The presence of minimal ETE in PTC has been associated with LN metastasis, local recurrence, and poor prognosis.^14^–^16^ However, recent studies have called into question the prognostic significance of the presence of minimal ETE for tumor recurrence in PTC.^17^–^19^ Tumors with minimal ETE can be classified as category T3, irrespective of tumor diameter, according to the AJCC TNM cancer staging system.^2^ Thus, most cases of PTMCs can reveal the presence of minimal ETE after microscopic evaluation, especially when tumors are peripherally located despite a small diameter (accounting for 43 % of cases in the present study). Moreover, the benefit of evaluating true capsular invasion to confirm minimal ETE has been debated among endocrine pathologists because the anatomy of the thyroid gland demonstrates no definite fibrous capsule. The adipose tissue and fibers of the skeletal muscle, which are usually observed in the extrathyroid area, are normally also present within the thyroid parenchyma.^10^,^11^ Although the involvement of skeletal muscle is a more reliable feature for confirming the presence of minimal ETE than involvement of the perithyroid soft tissue, skeletal muscle is not uniformly distributed throughout the thyroid gland and can even be mixed with thyroid follicles in the isthmus.^12^ Identification of thick extrathyroid arteries might be helpful for confirming the presence of minimal ETE, but there is no reliable anatomical landmark that can serve as a criterion for minimal ETE.^20^ In our present study, we microscopically subclassified the extent of minimal ETE to estimate and compare the effect on RFS based on extension to the perithyroid soft tissue or the sternothyroid muscle, respectively. We found that invasion to the sternothyroid muscle had no dominant effect on RFS (Table 2). Furthermore, our current analyses suggested that the current definition for minimal ETE based on the AJCC TNM cancer staging system (E0 vs. E1 + E2) revealed the most statistically significant difference in RFS.^2^

Among the various clinicopathological parameters that we tested, older age, larger tumor size, and the presence of LN metastasis were each found to be associated with the presence of minimal ETE (Table S3), but there was no significant difference in terms of gender. Although male patients exhibited a significantly higher recurrence rate (Table 3) for PTC, these estimates were limited by the relatively small number of male patients (11.4 %, 62/546). Moreover, there was no significant difference in PTMC found to be related to gender. The only independent prognostic factor that we found to be associated with recurrence was the presence of LN metastasis in our solitary PTC but not in solitary PTMC (Tables 3, 4). In cases with LN metastasis, presence of extranodal extension and macrometastasis was significantly associated with a higher recurrence rate. The reason why there was a significance in N stage in univariate analysis but no effect in multivariate analysis might be caused by an effect that the presence of extranodal extension and larger metastatic tumor size displayed tendency of higher N stage. The presence of minimal ETE had no effect on RFS in solitary PTC and PTMC regardless of types of surgery (Tables 3, 4; Fig. 1; Tables S1 and S5). This finding is consistent with the results of previous studies of RFS or relapse-free survival in both PTC and PTMC patient groups.^17^–^19^,^21^

Our current retrospective study had several limitations of note, including a small sample size (546 PTCs, 144 PTMCs). A larger study of RFS in patients with PTMCs needs to be performed in the future. Indeed, a larger multicenter study will be needed for the proper validation of our current findings. Various effects of different clinical treatment modalities, such as RAI, were not analyzed for RFS in our present study. We attempted to eliminate the effects of multifocal PTCs in RFS, which limited our analysis to cases with solitary PTCs.

## Conclusions

The presence of LN metastasis is the only independent prognostic factor associated with RFS in solitary PTC patients. The presence or absence of minimal ETE has no significant effect on RFS in solitary PTC and PTMC. Because the microscopic diagnostic criteria for the presence of minimal ETE remain the subject of debate, there is risk of over assigning patients with PTMC to the T3 category based on the present AJCC TNM cancer staging system.

## Electronic supplementary material

Supplementary material 1 (DOCX 24 kb)

Supplementary material 2 (PPTX 90 kb)
