# Supplementary material for: Clinicopathological Significance of Minimal Extrathyroid Extension in Solitary Papillary Thyroid Carcinomas
Source: Ann Surg Oncol. 2015 Jun 16;22:728–33. doi: 10.1245/s10434-015-4659-0 (PMC4686556; doi:10.1245/s10434-015-4659-0)
Supplement: Supplementary file 1 — Supplementary material 1 (DOCX 24 kb) [file 10434_2015_4659_MOESM1_ESM.docx]

Supplementary data

| Table S1. Characteristics of lymph node metastasis in solitary papillary thyroid carcinoma | |
| --- | --- |
| Lymph node metastasis | Number (%) |
| Metastatic tumor size^1^ |  |
| Mean size (mm, range) | 6.1 (0.1 - 38) |
| Isolated tumor cells (ITC) | 4 (1.2) |
| Micrometastasis | 111 (33.9) |
| Macrometastasis | 212 (64.8) |
| N stage |  |
| Central metastasis (N1a) | 277 (82.9) |
| Lateral metastasis (N1b) | 57 (17.1) |
| Extranodal extension^1^ |  |
| Absence | 216 (66.1) |
| Presence | 111 (33.9) |
| ^1^Total cases with lymph node metastasis were 334 cases, but there were 7 cases with missing blocks and slides. | |

| Table S2. The 12 recurrences in the previous resection sites (%) | | |
| --- | --- | --- |
| Recurrent site | operation bed | Contralateral lobe |
| Total thyroidectomy |  |  |
| Clear margin | 6 (50) | 0 (0) |
| Involved margin | 5 (41.7) | 0 (0) |
| Lobectomy |  |  |
| Clear margin | 0 (0) | 1 (8.3) |
| Involved margin | 0 (0) | 0 (0) |

| Table S3. Clinicopathologic parameters associated with the presence of minimal extrathyroid extension (ETE) in solitary papillary thyroid carcinoma | | | |
| --- | --- | --- | --- |
| Parameters | OR | 95% CI | *p* value |
| Age (yrs) |  |  |  |
| < 45 | 1 (ref) |  |  |
| ≥ 45 | 1.688 | 1.154 - 2.469 | 0.007 |
| Sex |  |  |  |
| Male | 1 (ref) |  |  |
| Female | 1.285 | 0.702 - 2.353 | 0.416 |
| Size (mm) |  |  |  |
| ≤ 10 | 1 (ref) |  |  |
| > 10 | 3.078 | 2.040 - 4.645 | <0.001 |
| Cervical Lymph node |  |  |  |
| No metastasis | 1 (ref) |  |  |
| Metastasis | 2.071 | 1.407 - 3.048 | <0.001 |

| Table S4. The extent of lymph node metastasis associated with recurrence of solitary papillary thyroid carcinomas | | | | | | | |  |
| --- | --- | --- | --- | --- | --- | --- | --- | --- |
| Lymph node metastasis | Univariate | | |  | Multivariate | | | |
|  | HR | 95% CI | *p* value |  | HR | 95% CI | *p* value | |
| N stage |  |  |  |  |  |  |  | |
| Central metastasis (N1a) | 1 (ref) |  |  |  | 1 (ref) |  |  | |
| Lateral metastasis (N1b) | 2.619 | 1.458 - 4.703 | 0.001 |  | 1.058 | 0.564 - 1.985 | 0.860 | |
| Extranodal extension |  |  |  |  |  |  |  | |
| Absence | 1 (ref) |  |  |  | 1 (ref) |  |  | |
| Presence | 5.009 | 2.734 - 9.177 | <0.001 |  | 3.474 | 1.853 - 6.512 | <0.001 | |
| Types |  |  |  |  |  |  |  | |
| Micrometastasis/ITC | 1 (ref) |  |  |  | 1 (ref) |  |  | |
| Macrometastasis | 6.801 | 2.448 - 18.897 | <0.001 |  | 4.052 | 1.403 - 11.706 | 0.011 | |

| Table S5. Minimal extrathyroid extension (ETE) associated with recurrence of solitary papillary thyroid carcinomas according to types of surgery (multivariate analysis). | | | | | | | |
| --- | --- | --- | --- | --- | --- | --- | --- |
| Parameters | Lobectomy/less than total resection | | |  | Total thyroidectomy | | |
|  | HR | 95% CI | *p* value |  | HR | 95% CI | *p* value |
| Invasion |  |  |  |  |  |  |  |
| Confinement | 1 (ref) |  |  |  | 1 (ref) |  |  |
| Minimal ETE | 1.114 | 0.101 - 12.289 | 0.930 |  | 1.782 | 0.898 - 3.533 | 0.098 |

**Figure legend**

**Figure S1.** Recurrence-free survival (RFS) according to subclassification of minimal extrathyroid extension in solitary PTCs. A more significant difference in the RFS was found in two-group (*p*=0.006, E0 versus E1+E2; B) versus three-group (*p*=0.021, E0 versus E1 versus E2;A) comparisons using both Kaplan–Meier analysis and the log rank test. Another two-group comparison (E0+E1 versus E2,C) showed no significant difference (*p*=0.686).
