# Supplementary material for: Clinicopathological Significance of Minimal Extrathyroid Extension in Solitary Papillary Thyroid Carcinomas
Source: Ann Surg Oncol. 2015 Jun 16;22:728–33. doi: 10.1245/s10434-015-4659-0 (PMC4686556; doi:10.1245/s10434-015-4659-0)
Supplement: Supplementary file 2 — Supplementary material 2 (PPTX 90 kb) [file 10434_2015_4659_MOESM2_ESM.pptx]

## Slide 1
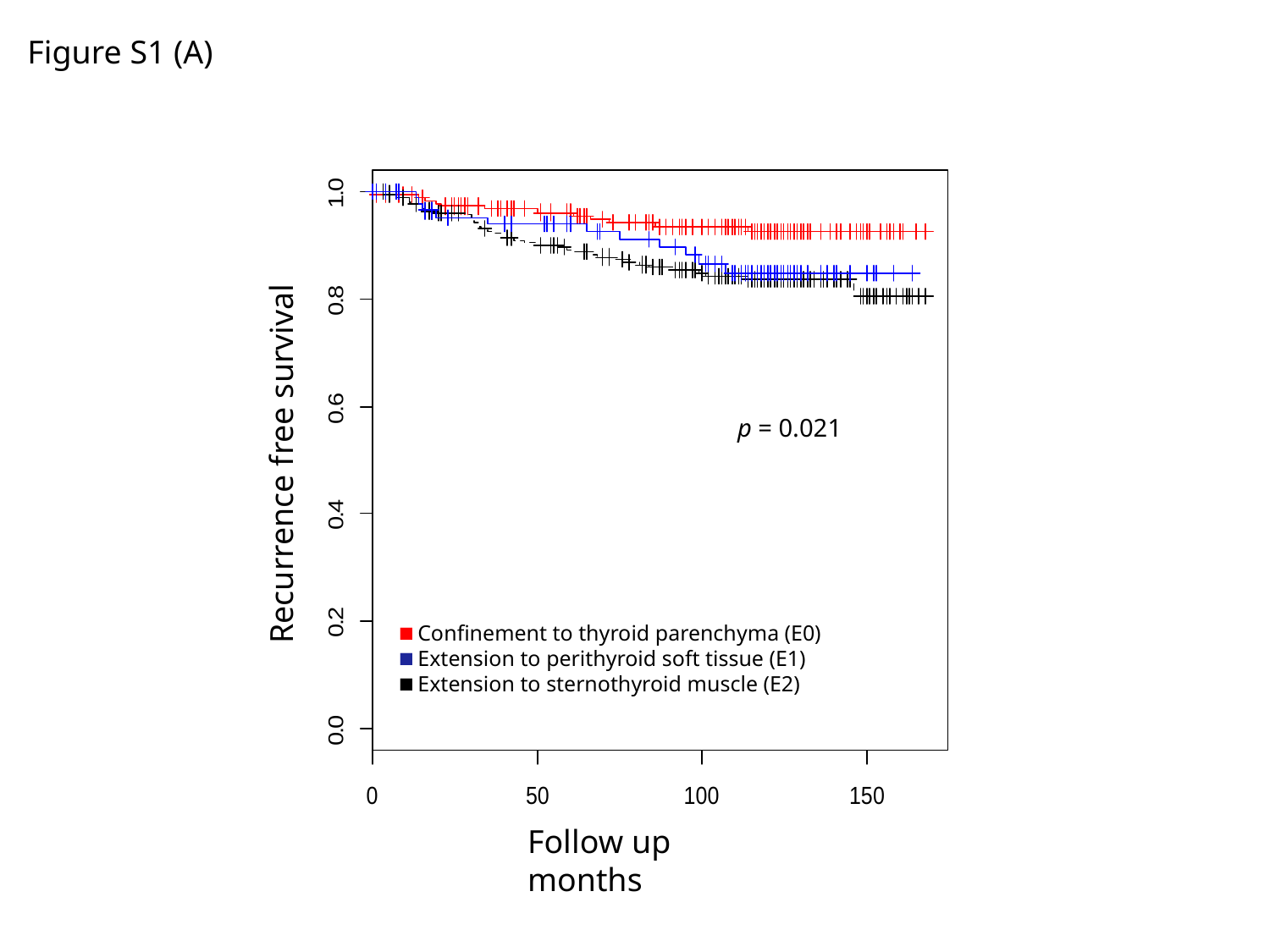

Figure S1 (A)
p = 0.021
Recurrence free survival
 Confinement to thyroid parenchyma (E0)
 Extension to perithyroid soft tissue (E1)
 Extension to sternothyroid muscle (E2)
Follow up months

## Slide 2
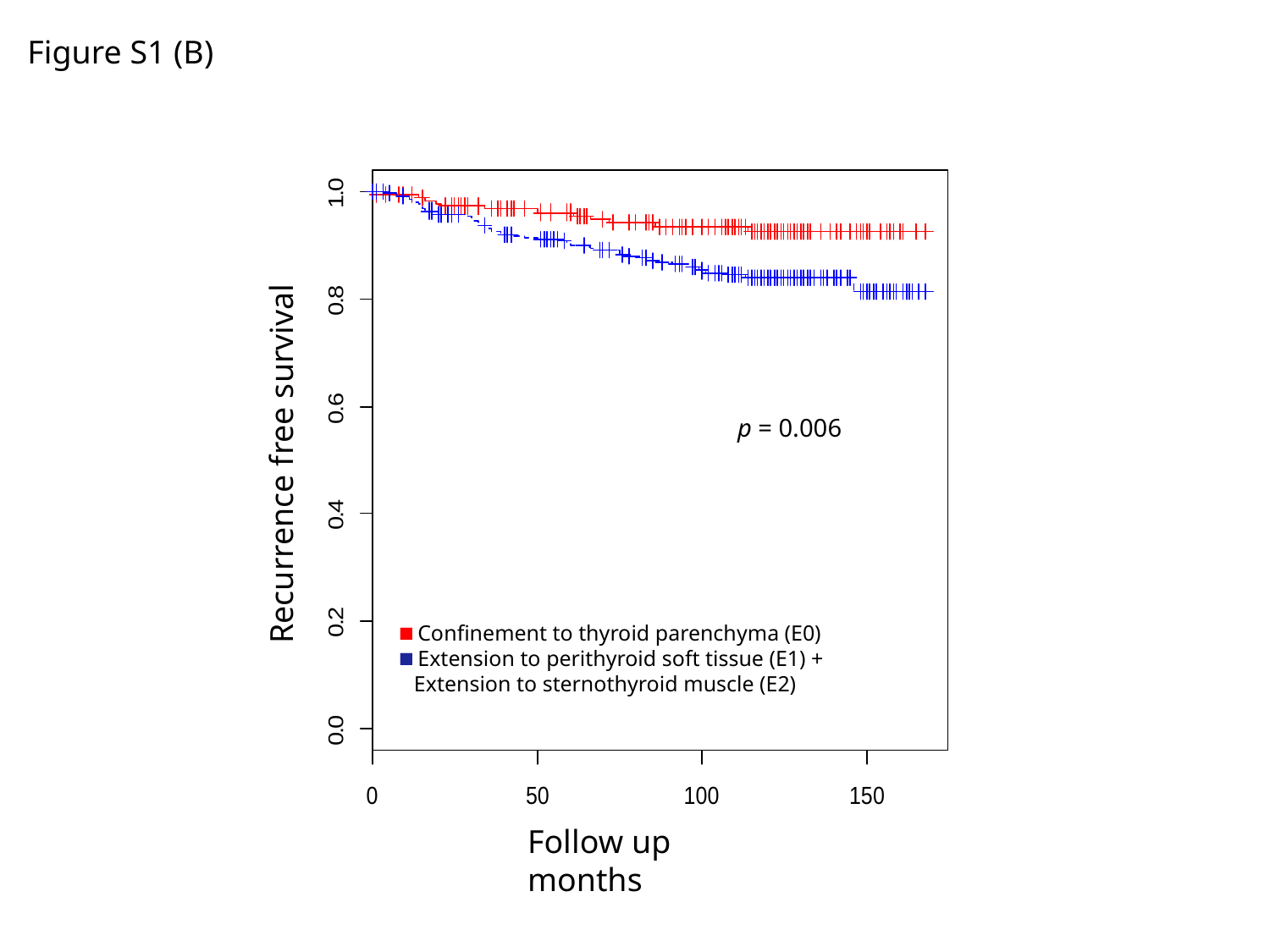

Figure S1 (B)
p = 0.006
Recurrence free survival
 Confinement to thyroid parenchyma (E0)
 Extension to perithyroid soft tissue (E1) +
 Extension to sternothyroid muscle (E2)
Follow up months

## Slide 3
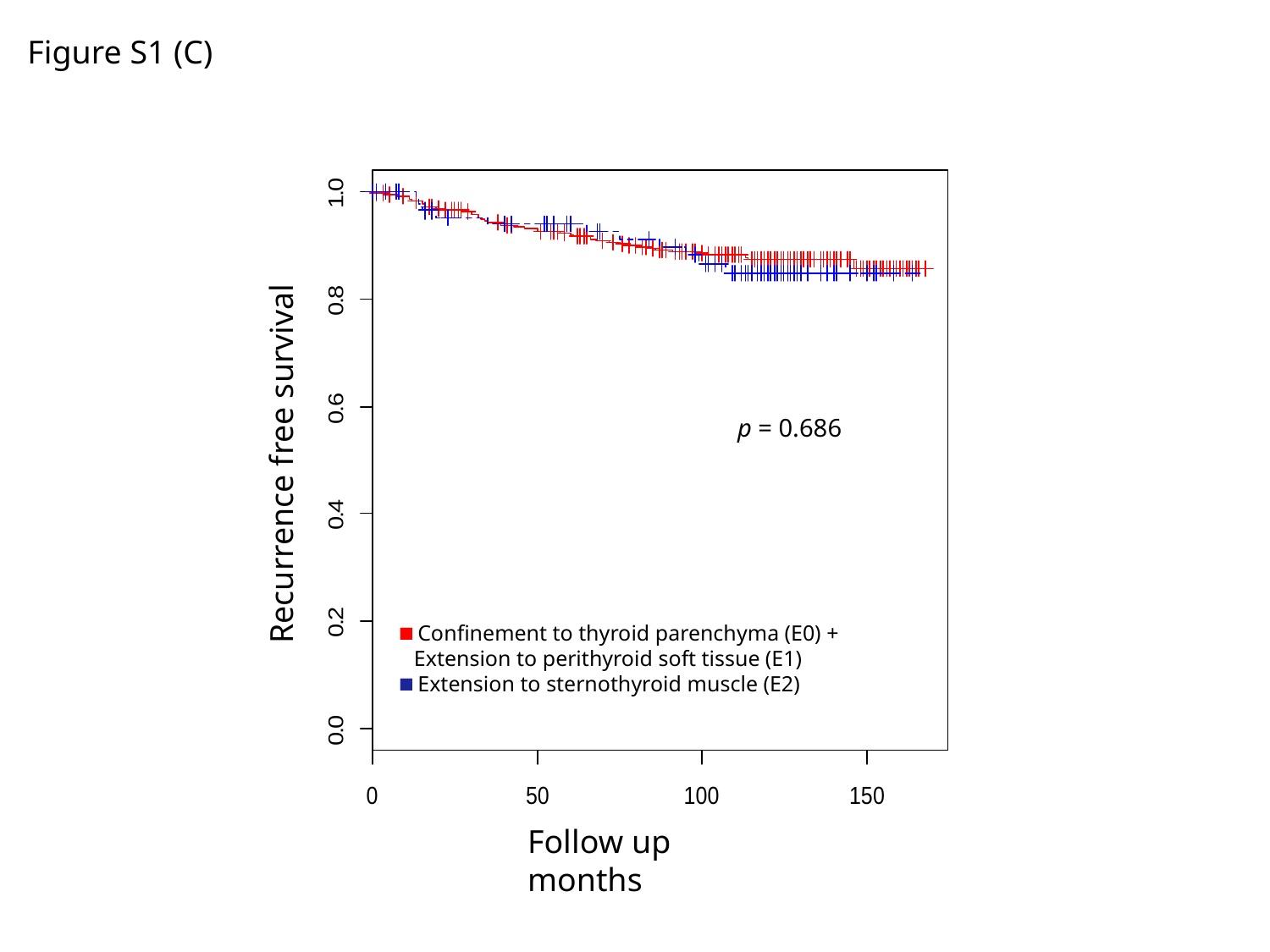

Figure S1 (C)
p = 0.686
Recurrence free survival
 Confinement to thyroid parenchyma (E0) +
 Extension to perithyroid soft tissue (E1)
 Extension to sternothyroid muscle (E2)
Follow up months
